# Use of artificial intelligence in the management of stroke: scoping review

**DOI:** 10.3389/fradi.2025.1593397

**Published:** 2025-05-23

**Authors:** Nicolas Melo Sierra, Erwin Hernando Hernández Rincón, Gabriela Alejandra Osorio Betancourt, Paula Andrea Ramos Chaparro, Diana Marcela Diaz Quijano, Samuel David Barbosa, Michel Hernandez Restrepo, Gustavo Uriza Sinisterra

**Affiliations:** ^1^ Faculty of Medicine, Universidad de La Sabana, Chía, Colombia; ^2^ Department of Family Medicine and Public Health, Universidad de La Sabana, Chía, Colombia; ^3^ Department of Epidemiology, Universidad de la Sabana, Chia, Colombia; ^4^ Faculty of Medicine, Universitat Oberta Catalunya, Barcelona, Spain; ^5^ Department of Radiology, Hospital Universitario de La Samaritana, Bogotá, Colombia; ^6^ Department of Neurosurgery, Clínica Universidad de La Sabana, Chía, Colombia

**Keywords:** artificial intelligence, ischemic stroke, hemorrhagic stroke, primary health care, machine learning algorithms

## Abstract

**Introduction:**

Stroke is a condition that is more predominant in developed countries. However, it continues to be considered a high-cost health pathology worldwide, both in the medium and long term. Therefore, diagnosis, treatment, and rehabilitation are vital. Additionally, the assistance of artificial intelligence in these three principles has been increasing, given its effectiveness and efficiency in performance.

**Objective:**

This study analyzes the available evidence regarding the use of artificial intelligence in primary care for stroke patients.

**Methods:**

A scoping review was conducted on three indexed databases, Science Direct, Web of Science, and PubMed, resulting in the identification of 1,382 articles. Initially, these terms were filtered on the basis of the year of publication and language. A second distinction was subsequently made through the title and abstract of each publication.

**Results:**

A total of 33 articles summarizing 5 categories were selected: healthcare from a general point of view; stroke prediction; the diagnosis and treatment of both stroke and its sequelae; the risk of death in the poststroke period; and the assistance of AI in some specialties related to the disease.

**Conclusion:**

Artificial intelligence has the potential to improve stroke care, but more research is still needed to evaluate its performance in clinical practice.

**Introducción:**

El accidente cerebrovascular es una condición predominante en los países desarrollados. A pesar de esto, es una patología de salud de alto costo en todo el mundo, tanto a mediano como a largo plazo. Por lo tanto, el diagnóstico, el tratamiento y la rehabilitación son de vital importancia. Por lo anterior, la asistencia de la inteligencia artificial en estos tres principios ha ido en aumento, dada su eficacia y eficiencia en el desempeño.

**Objetivo:**

Este estudio analiza la evidencia disponible sobre el uso de la Inteligencia Artificial en la atención primaria para el accidente cerebrovascular.

**Métodos:**

Se realizó una revisión tipo Scoping Review en tres bases de datos indexadas: Science Direct, Web of Science y PubMed, lo que resultó en la identificación de 1,382 artículos. Inicialmente, estos se filtraron en función del año de publicación y el idioma. Posteriormente, se realizó una segunda distinción a través del título y el resumen de cada publicación.

**Resultados:**

Se seleccionaron un total de 33 artículos, que se seleccionaron en 5 categorías: atención médica desde un punto de vista general; predicción de accidente cerebrovascular; diagnóstico y tratamiento tanto del accidente cerebrovascular como de sus secuelas; riesgo de muerte en el período posterior al accidente cerebrovascular; y finalmente, la asistencia de la Inteligencia Artificial en algunas especialidades relacionadas con la enfermedad.

**Conclusión:**

La Inteligencia Artificial tiene el potencial de mejorar la atención del accidente cerebrovascular, pero aún se necesitan más investigaciones para evaluar su desempeño en la práctica clínica.

## 1 Introduction

A stroke is a condition that affects the arteries supplying or located within the brain; it occurs when a blood clot significantly restricts the artery's caliber or when the artery ruptures, disrupting the transport of oxygen and nutrients to the brain and consequently leading to cell death (1). At the global level, there is an estimated age-standardized incidence rate of ischemic stroke, the most common subtype, at 89.32 per 100,000 inhabitants in 2030, with an estimated annual percentage change of 0.89. Additionally, a greater increase in the incidence rate is projected for women than for men in 2030 (90.70 vs. 87.64 per 100,000). In conclusion, the incidence of ischemic stroke is expected to increase in both sexes, showing a general trend worldwide from 2020–2030 (2).

As a condition incurring high treatment costs in the medium and long term, early diagnosis and treatment are highly important. Various recognition strategies based on symptoms, such as F.A.S.T. (Face; Arms; Speech; Time) (3) or Recognition of Stroke in the Emergency Room (ROSIER), which are other tools that evaluate loss of consciousness or syncope, seizures, asymmetric facial weakness, asymmetric arm weakness, asymmetric leg weakness, speech impairment, and visual field defects (4), have been implemented for immediate recognition. Intrahospital guidelines for the initial management of stroke recommend a simple computerized tomography (CT) scan as the first imaging procedure in the emergency department, followed by scales such as the Alberta Stroke Program Early Computed Tomography Score (ASPECTS) or other more specialized neuroimaging methods, such as CT-Angiogram, CT perfusion, or nuclear magnetic resonance (5).

On another note, the assistance of artificial intelligence (AI), understood as a branch of informatics seeking to understand how intelligent entities function to create software programs simulating their operation, has gained momentum in the healthcare sector over the last decade. Since the COVID-19 pandemic, it has become a topic of interest in the scientific community as a means of supporting the workload of healthcare workers (6, 7). AI has expanded to various applications; initially, early models aimed to support clinical decisions through the analysis of specific tasks such as interpreting electrocardiogram signals, disease diagnosis, selecting appropriate treatments, and interpreting clinical reasoning.

Currently, the use of AI has expanded into health service management, predictive medicine, and data management. The two most commonly used instruments in healthcare applications are machine learning (ML) and deep learning (DL). ML algorithms can perform specific tasks by recognizing relationships in collected data, whereas DL, with its numerous hidden internal layers, can store vast datasets. Despite their high productivity demonstrated in the healthcare sector, they have been perceived as “black boxes,” especially in processes such as prediction and result verification. Therefore, explainable AI has been introduced as a technique to increase confidence in predictions, promoting the use of these new tools (7). Medical specialties such as neurosurgery and radiology are at the forefront of the use and implementation of AI in their daily work, which is sometimes hindered by socioeconomic or adaptational factors (8).

In this context, an investigation was conducted to analyze the available evidence regarding the use of AI in primary care for stroke patients. Importantly, current AI studies in stroke patients have focused primarily on acute care, whereas chronic management and primary care have been largely overlooked—highlighting the relevance and necessity of this study.

## 2 Material and methods

A scoping review of the existing scientific literature was conducted with the objective of identifying the type of evidence available in the field of stroke research and its integration with AI. The study defined the population as patients diagnosed with hemorrhagic or ischemic stroke, the concept as the application of AI, and the context as primary care settings.

The review utilized indexed electronic databases, including Science Direct, Web of Science, and PubMed, to retrieve relevant studies. MeSH and DeCS terms such as “Hemorrhagic stroke,” “Ischemic stroke,” “Primary care,” and “Artificial intelligence” were employed to refine the search strategy. An initial pool of 1,382 articles was identified.

**Figure 1:**
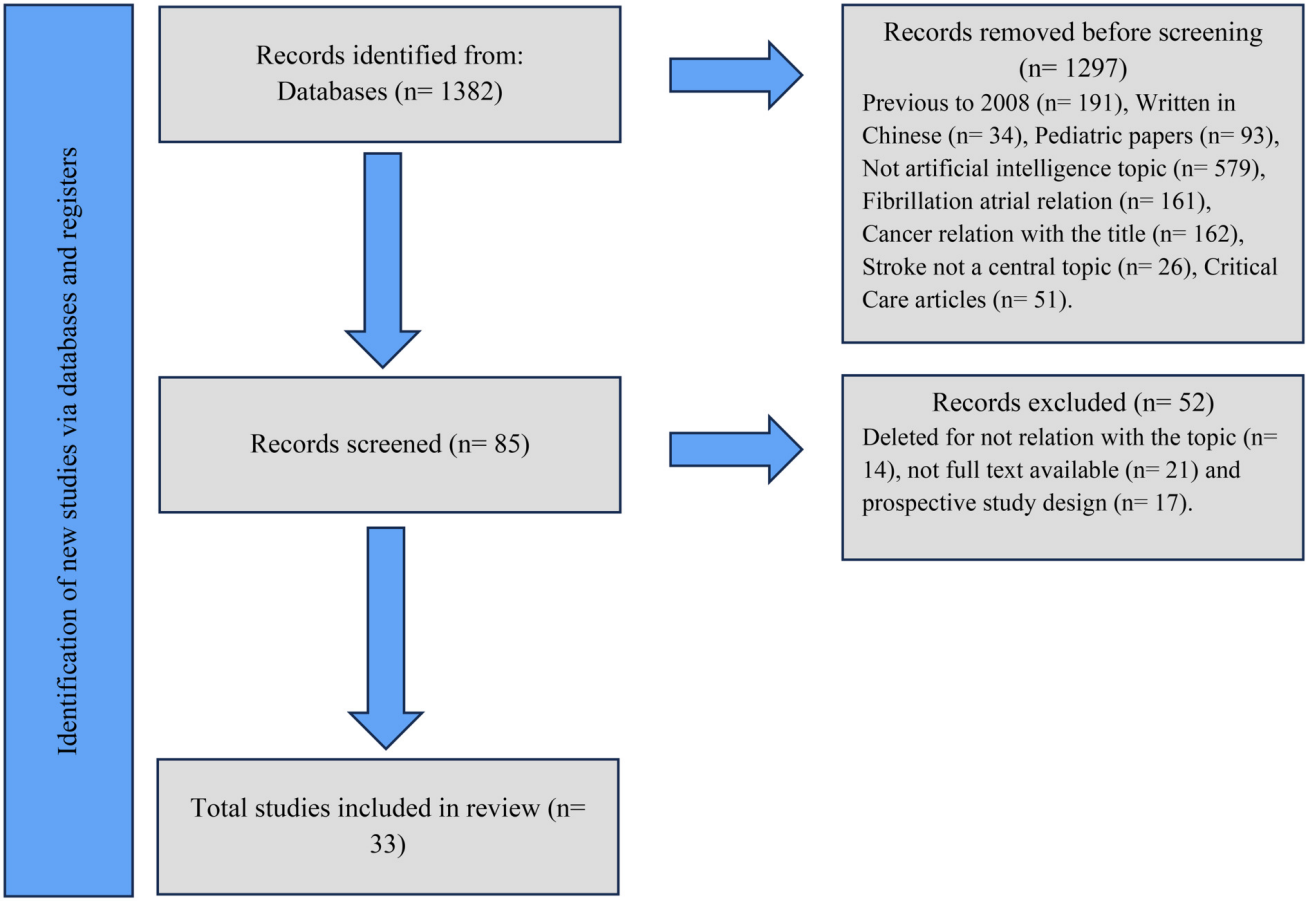
Flow diagram showing the systemic review process.

The review spanned a timeframe from 2008–April 2024, focusing on publications in English, Portuguese, and Spanish. Articles were selected on the basis of eligibility criteria, which emphasized relevance to the intersection of primary care, stroke management, and AI. The methodological quality of the included studies was assessed and organized following the PRISMA checklist, as presented (Figure 1). The review included diverse publication types, such as meta-analyses, scoping reviews, literature reviews, bibliometric analyses, retrospective cross-sectional studies, and studies involving big data.

In contrast, exclusion criteria ruled out prospective studies, case reports, gray literature, and studies focusing on animals, pediatrics, cadavers, cancer, atrial fibrillation, or stroke presentations attributable to known pathological causes.

The selection process began with a preliminary screening on the basis of article titles, followed by an abstract review to ensure relevance to the study's objectives. Articles that passed this stage underwent a more thorough analysis. To facilitate data organization, a Microsoft Excel table was created, categorizing the publications by their focus and documenting detailed information such as the author, title, year of publication, and abstract. This comprehensive review process included a detailed reading of each selected article.

The methodology was further strengthened by supervision from three independent reviewers, who collaborated through Microsoft Word Outline documents and PowerPoint presentations. Discrepancies encountered during the process were discussed and resolved in eight meetings, ensuring consensus without conflicts of interest.

From the initial search, 1,157 relevant articles were identified on the basis of the established criteria. However, 1,072 articles were excluded for reasons such as duplication, stroke was not the main focus, or the study was not related to the field of AI. This left 85 articles, which were subjected to a full-text review via a more refined approach to the exclusion criteria, as there was only a minimal connection between stroke and AI. The full text was not available despite multiple attempts to contact the author or obtain it through interlibrary loan, and/or the study had a prospective design.

Ultimately, 33 articles were included in the final analysis. These were classified into five thematic categories:

1. General medical care: 2 articles.
2. Stroke prediction: 2 articles.
3. Diagnosis and Treatment of Stroke and Its Sequelae: 23 articles.
4. Risk of death after a stroke: 3 articles.
5. AI in specialties related to stroke: 3 articles.

This categorization is summarized (Table 1) and provides a clear framework for understanding the focus areas and applications of AI in stroke care and management.

**Table 1:** Methodology for references.

N°	Authors	Title	Year	Key findings	Population identified	Type of study
The application of AI in the healthcare field:						
1	Jimma, Bahiru Legesse, and Cols.	Artificial intelligence in healthcare: A bibliometric analysis	2023	Artificial Intelligence related to healthcare involves understanding its growth and requirements for the responsible use of this new tool.	No specific clinical population.	Bibliometric analysis
2	Habuza, Tetiana, and Cols.	AI applications in robotics, diagnostic image analysis and precision medicine: Current limitations, future trends, guidelines on CAD systems for medicine	2021	The challenges and benefits of Artificial Intelligence and robotics in healthcare.	No specific clinical population.	Systematic review
Use of AI for predicting the occurrence of a stroke:						
3	Maharjan J and Cols.	Enriching the Study Population for Ischemic Stroke Therapeutic Trials Using a Machine Learning Algorithm.	2021	Learning algorithms as a more precise tool for predicting the near-term risk of presenting an ischemic stroke, within a prediction window of 1 year.	Patients with ischemic stroke	Retrospective observational study
4	Sirsat, Manisha Sanjay, and Cols.	Machine Learning for Brain Stroke: A Review	2020	The efficiency of machine learning in fast and accurate prediction in personalized clinical care for stroke patients.	Stroke patients	Systematic review
Diagnosis and treatment based on the type of stroke with the assistance of Artificial Intelligence:						
5	Delio PR, and Cols.	Assistance from Automated ASPECTS Software Improves Reader Performance.	2021	Comparison between ASPECTS interpretation in patients with large vessel occlusion assisted by automatic software vs. without assistance.	No specific clinical population.	Observational study
6	Hassan, AE, and Cols.	The implementation of artificial intelligence significantly reduces door-in-door-out times in a primary care center prior to transfer	2022	Use of Artificial Intelligence as a tool for detecting large vessel occlusion through Computerized Tomography- Angiography, reducing the time interval from admission to discharge within the primary care center before transfer to the comprehensive care center.	Stroke patients	Retrospective cohort study
7	Gunda, B, and Cols.	Improved Stroke Care in a Primary Stroke Centre Using AI-Decision Support	2022	The classification of candidates for reperfusion therapies in stroke patients through machine learning.	Stroke patients	Retrospective cohort study
8	Al-Kawaz, M and Cols.	Impact of RapidAI mobile application on treatment times in patients with large vessel occlusion	2022	Utilization of the mobile application RápidAI as a rapid access tool for perfusion images in patients with large vessel occlusion to reduce times in the care and treatment of Stroke.	Patients with large vessel occlusions	Retrospective cohort study
9	Eldaya, RW, and Cols.	Performance of Automated RAPID Intracranial Hemorrhage Detection in Real-World Practice: A Single-Institution Experience	2022	The use of RAPID ICH software as a tool for automatic detection of intracranial hemorrhage through noncontrast Computerized Tomography in the emergency context.	Patients who presented with acute neurological symptoms suspicious for stroke	Retrospective cohort study
10	Karthik, R. and Cols.	Neuroimaging and deep learning for brain stroke detection—A review of recent advancements and future prospects	2020	Emphasizing the impact of deep learning algorithms in the detection of strokes and lesion segmentation.	No specific clinical population.	Systematic review
11	Salman, Saif, and Cols.	Artificial intelligence and machine learning in aneurysmal subarachnoid hemorrhage: Future promises, perils, and practicalities	2023	The use of Artificial Intelligence through machine learning for medical care of patients with aneurysmal subarachnoid hemorrhage based on presentation and severity.	No specific clinical population.	Systematic review
12	El Naamani, Kareem, and Cols.	The Artificial Intelligence Revolution in Stroke Care: A Decade of Scientific Evidence in Review	2024	The influence that Artificial Intelligence has had over the last decade on the care of patients with acute stroke.	No specific clinical population.	Systematic review
13	Bouazizi, Samar, and Cols.	Enhancing accuracy and interpretability in EEG-based medical decision making using an explainable ensemble learning framework application for stroke prediction	2024	Machine learning, coupled with data obtained from electroencephalograms, has become essential for disease prediction and cognitive assessment in the context of stroke.	No specific clinical population.	Methodological research study
14	Shafaat, Omid, and Cols.	Leveraging artificial intelligence in ischemic stroke imaging	2022	The application of Artificial Intelligence in the diagnostic and clinical treatment of stroke directed toward physicians.	Clinicians involved in stroke management	Review article
15	Yoon, Chiho, and Cols.	Collaborative multimodal deep learning and radiomic features for classification of strokes within 6 h	2023	Deep learning method for classifying acute stroke within 6 h and determining the need for endovascular thrombectomy.	No specific clinical population.	Methodological study
16	Gupta, Rajiv, and Cols.	An East Coast Perspective on Artificial Intelligence and Machine Learning: Part 1: Hemorrhagic Stroke Imaging and Triage	2020	The performance of Artificial Intelligence in the detection, segmentation, quantification, and treatment of hemorrhagic stroke.	No specific clinical population.	Systematic review
17	Ghozy S; and Cols.	The diagnostic performance of artificial intelligence algorithms for identifying M2 segment middle cerebral artery occlusions: A systematic review and meta-analysis.	2023	Artificial Intelligence-based algorithm to facilitate the diagnostic performance of stroke due to occlusions of medium-sized vessels (M2)	No specific clinical population.	Systematic review
18	Salman, Saif and Cols.	Hemorrhage Evaluation and Detector System for Underserved Populations: HEADS-UP	2023	Utilization of Artificial Intelligence through rapid machine learning is a quick method for the detection of intracranial hemorrhages in resource-limited hospitals.	No specific clinical population.	Retrospective cohort study
19	Nafees Ahmed, S. and Cols.	A systematic review on intracranial aneurysm and hemorrhage detection using machine learning and deep learning techniques	2023	Study of multiple challenges in the detection of hemorrhages and aneurysms through automatic models and deep learning.	Individuals at risk of developing intracranial aneurysms and subarachnoid hemorrhage	Observational study
20	Luo, Wenmiao, and Cols.	The Influence of the Novel Computer-Aided Triage System Based on Artificial Intelligence on Endovascular Therapy in Patients with Large Vascular Occlusions: A Meta-Analysis	2023	The novel impact of the Artificial Intelligence-assisted computer-based classification system (AI-CTS) on endovascular therapy in patients with large vessel occlusions is examined.	Patients with large vascular occlusions	Systematic review
21	Kunst, Mara and Cols.	Real-World Performance of Large Vessel Occlusion Artificial Intelligence–Based Computer-Aided Triage and Notification Algorithms—What the Stroke Team Needs to Know	2023	Clinical performance of two FDA-approved Artificial Intelligence-based computer-aided detection, classification, and notification devices (CADt) in the context of stroke.	Patients undergoing “code stroke” CT angiography examinations	Retrospective study
22	Martinez-Gutiy and Cols.	Automated Large Vessel Occlusion Detection Software and Thrombectomy Treatment Times: A Cluster Randomized Clinical Trial.	2023	Use of automated software for the detection and classification of large vessel occlusion with Artificial Intelligence in relation to the benefit of endovascular thrombectomy.	Patients with large vessel occlusion	Cluster randomized stepped-wedge clinical trial
23	Yedavalli, Vivek S, and Cols.	Artificial intelligence in stroke imaging: Current and future perspectives	2021	Performance of Artificial Intelligence through supervised and deep machine learning in terms of image acquisition, reconstruction, and interpretation of images in patients with acute stroke.	Patients with acute stroke	Systematic review
24	Meng, Shujuan, and Cols.	End-to-end artificial intelligence platform for the management of large vessel occlusions: A preliminary study	2022	Deep learning detecting large vessel occlusion and predicting functional outcome based on Computerized Tomography-Angiography.	Patients with large vessel occlusion	Retrospective study
25	Liu, Yuzhe, and Cols.	Big Data in Stroke: How to Use Big Data to Make the Next Management Decision	2023	The use of Artificial Intelligence for classifying patients who benefit from thrombolytic interventions or mechanical thrombectomy through macro data.	Patients who have experienced a stroke	Narrative review
26	Gupta, Rajiv, and Cols.	An East Coast Perspective on Artificial Intelligence and Machine Learning: Part 2: Ischemic Stroke Imaging and Triage	2020	In this second part, Artificial Intelligence techniques for the management of strokes are analyzed	No specific clinical population.	Systematic review
27	Ma, Yang, and Cols.	Artificial intelligence: The dawn of a new era for cutting-edge technology-based diagnosis and treatment for stroke	2020	The application of AI in the four main areas of stroke care: diagnosis, prediction of sequelae, treatment, and rehabilitation.	Patients with stroke	Systematic review
Use of AI to predict the risk of death following a stroke:						
28	Schwartz, Lihi, and Cols.	Stroke mortality prediction using machine learning: systematic review	2023	Machine learning algorithms to predict the sequelae of strokes.	Patients who have suffered a stroke	Systematic review
29	Ho KC; and Cols.	Predicting discharge mortality after acute ischemic stroke using balanced data	2014	The use of software for predicting mortality in stroke patients at the time of hospital discharge.	Stroke patients	Comparative analysis
30	Lin CH and Cols.	Development and Validation of a Novel Score for Predicting Long-Term Mortality after an Acute Ischemic Stroke	2023	A prediction model to identify patients at risk of long-term mortality after a stroke.	Patients with acute ischemic stroke	Retrospective cohort study
AI assistance in some specialties:						
31	El-Hajj, Victor Gabriel and Cols.	Artificial Intelligence in Neurosurgery: A Bibliometric Analysis	2023	The use of Artificial Intelligence in the field of neurology as a tool for diagnosis, treatment, and imaging interpretation.	No specific clinical population.	Bibliometric analysis
32	Vinny, P.W, and Cols.	Artificial Intelligence shaping the future of neurology practice	2021	Impact of Artificial Intelligence in the neurological field.	Neurologists and clinical practitioners	Overview
33	Duong, Michael Tran, and Cols.	Diverse Applications of Artificial Intelligence in Neuroradiology	2020	Diverse applications of Artificial Intelligence in neuroradiology.	Patients with brain tumors, stroke, trauma, multiple sclerosis, epilepsy, and neurodegenerative diseases.	Review article

## 3 Results

### 3.1 The application of AI in the healthcare field

AI, as previously mentioned, is a subdomain of computer science responsible for the creation of intelligent systems comparable to the intelligence of human behavior and reasoning. Although this term has been used exponentially, to date, there is no widely accepted definition of the term itself. In the last decade, this technology has had a major influence on human life given the different applications, such as engineering and health care, among other skills, that it has acquired (9).

The AI-based healthcare system enhances prediction, diagnosis, and medical interventions, benefiting both patients and healthcare professionals in four key areas: (1) It estimates treatment success probabilities and analyzes disease onset before treatment initiation; (2). It prevents or manages complications; (3) It actively supports patient care during diagnosis and/or treatment; and (4) Pathology can be identified, and the best treatment can be selected. Additionally, AI has been found to help patients become aware of their condition even when complex terms are used, thus improving their quality of life and promoting greater treatment adherence. An example of this is AI, along with the internet of Medical Things (IoMT), where mobile applications can be used at any time to ask medical questions in a user-friendly manner, thus promoting patient awareness of their condition (9, 10).

In the last few decades, AI has developed across all branches of medicine, spanning from primary care to rare diseases, from emergency medicine to biomedical research and aspects related to public health (9). While diagnostic disciplines such as pathology and radiology have been particularly prominent, AI has also been utilized in areas such as neurosurgery, dermatology, gastroenterology, and cardiology. However, these tools are still in the research phase and have not been fully implemented in clinical practice (9, 10).

Two systems have recently begun to be incorporated: the “Idx-DR system,” which is software designed for diagnosing diabetic retinopathy, and “Viz. AI,” which assists in diagnosing patients with strokes through CT scans. Another example of successful collaboration through AI is Google's “DeepMind Health,” where researchers, physicians, and patients collaborate to address questions regarding real-world healthcare via ML algorithms represented in neural networks designed to mimic the human brain (9, 10).

On the other hand, image analysis is another of the most recognized applications of this new instrument, where AI promises to improve detection on the basis of tissue characterization and has demonstrated exceptional precision in the identification of anomalies. In the pictures (9). Currently, attempts have been made to implement “computer-aided detection (CAD) systems”, which have been shown to help expert fatigues in reading images; however, their use remains controversial, given that studies that have not shown improvements in the performance of radiologists in daily practice have been conducted (10).

It is estimated that by 2030, chronic diseases constitute 80% of all diseases, generating a high burden of morbidity worldwide, which is why researchers have concentrated their studies and efforts on the early detection and management of conditions via this type of advanced technology (9).

Another benefit attributed to AI is in the field of pharmacology. According to the California Biomedical Research Association, it takes approximately 12 years for a drug to reach the market, but with the assistance of these new technologies, this time could be significantly reduced. Additionally, AI can comprehend the drug's principal bioavailability and, on the basis of data obtained from a specific patient, can establish reliable biomarkers and molecules, thereby reducing costs and, as mentioned earlier, significantly shortening the time to market (10).

### 3.2 Use of AI to predict stroke

Strokes represent the second cause of mortality worldwide and are associated with long-term disability, with increased costs in care both at the time of diagnosis and subsequent disability, generating a significant impact on the quality of life of people who suffer from it. The risk factors for stroke are divided into 2 groups: nonmodifiable factors, such as being female, being over 55 years old, being in an ethnic-racial minority, and modifiable factors, which are related to physical activity, obesity, smoking, some comorbidities, and isolation (11).

AI together with ML are tools that have been studied as useful future technologies for the prediction of this type of disease, with a focus on the selection of patients at high and not substantial risk of suffering from it. Maharjan J et al. carried out a study with 715,836 patients who had previous hospitalizations, and from the data obtained from the selected sample, they developed a tree classifier-type method, which would predict ischemic stroke in a time period of less than 12 months, using the “Extreme Gradient Boosting” (XG Boost v1.3.3) in Python (v3.6.13) to implement a decision tree model based on certain characteristics extracted from the patients selected for this study; they achieved an area under the receiving operating characteristic (AUROC) curve of 0.880 (95% CI [0.873–0.879] for the prediction of ischemic stroke. The control classification was the “CHA2DS2 VASc”, achieving an AUROC of 0.7565 (95% CI [0.7331–0.7569]); with respect to the sensitivity and specificity of the XG Boost algorithm, 80% and 74%, respectively, were obtained, indicating high performance of the tool in stroke prediction up to 1 year after hospitalization (11).

Another example that has recently been studied is the subtypes of ML. Researchers Sirsat, Manisha Sanjay, et al., developed an article based on 39 publications regarding the contribution of ML in addressing some issues in stroke care. They describe the three most studied ML subtypes in this pathology thus far, which are “supervised learning,” where software assigns an input to an output on the basis of observations and predicts the output. Second, “unsupervised learning,” which involves grouping observations and forming clusters on the basis of their similarity, is used. Finally, DL involves developing a computational model with layers of processing to progressively store data from raw inputs (12).

On the other hand, in image interpretation, two DL architectures are used: “Convolutional Neural Networks” (CNNs) and “Recurrent Neural Networks” (RNNs), which focus on problem solving when processing images. On the basis of the above, it has been possible to study and partially understand the different uses of DL and ML, assisting in clinical trials focused on stroke care, and a higher accuracy of 99.3% in prediction was obtained. of ACVs through “spiking neural networks” (SNNs). Similarly, an accuracy of almost 100% was achieved for the classification of ischemic stroke subtypes via the “optimum path forest” (OPF) classifier with analysis of “brain tissue density” (ABTD) (12). In conclusion, the previously mentioned technologies are tools that are currently being studied, but they promise great advances in the approach and especially in the prediction of this disease, thus reducing the waste of resources and delay in starting treatment.

### 3.3 Diagnosis and treatment according to the type of stroke with the assistance of AI

Stroke is an acute neurological event that causes long-term disability and increases patient morbidity and mortality, making timely treatment crucial. Consequently, prioritizing image interpretation and optimizing workflows by calling relevant healthcare professionals have been among the most significant focuses of AI. For example, in the context of workflow organization optimization, Titano et al. utilized 3D convolutional neural networks prioritizing early CT scans with a high probability of neurological emergencies, including stroke, through a DL-type algorithm capable of interpreting and alerting doctors about urgent findings 150 times faster than did humans. On the other hand, as an example related to image diagnosis and interpretation, Prevedello et al. created a traditional ML algorithm with a sensitivity of 62% and a specificity of 96% for stroke detection via CT scans, yielding results similar to those of other studies and manual interpretation by neuroradiologists (13).

Another model for ischemic stroke detection has been Xception, which is part of a convolutional neural network that detects dense MCA signs with an accuracy of 86.55% (14). Additionally, as an example of explainable AI, the Ensemble Echo State Networks (E-ESN) model integrates data from intelligent support systems and interpretive knowledge of electroencephalograms provided by a physician, promoting the credibility of these technologies and likewise achieving better clinical outcomes. This model improved the stroke classification accuracy to 96.5% (15).

On the other hand, the treatment of stroke, especially the selection of candidates for mechanical thrombectomy or thrombolytic use, has been one of the most significant gaps in ischemic stroke management. To address this issue, big data, which is software that stores and analyzes published information on a specific topic, has been utilized. With this tool, models have been created to address these gaps. An example of this is the ML software package “TensorFlow,” created by Google Brain in 2015, which has served as the basis for multiple algorithms responsible for overseeing big data. Some of these algorithms are used for identifying additional urgent interventions, standardizing the detection of large vessel occlusion, and predicting the location and risk of hemorrhagic transformation. This is aimed at providing relevant information to aid decision-making (16).

Additionally, Gupta, Rajiv presented the results of 5 clinical trials, “MR CLEAN,” “ESCAPE,” “REVESCAT,” “EXTEND IA,” and “SWIFT-PRIME,” demonstrating the benefit of mechanical thrombectomy over the best medical treatment, including IV tPA when appropriate, extending the proposed window from 4.5 h from 2008 to 6 h after symptom onset. Similarly, it was demonstrated through a DL-type model, “multimodal U-Net” (MM-UNet), which is based on the analysis of images from a multimodal magnetic resonance. The performance of mechanical thrombectomy has been shown to be feasible up to 24 h after the onset of clinical symptoms in the context of proximal large vessel occlusion (LVO) (15,17,18). With respect to adherence to treatment, Laboviz et al. evaluated the use of AI-based mobile platforms to improve adherence to anticoagulant therapy in stroke patients. The study results revealed a significant improvement in adherence compared with patients who did not use the platform (19).

Furthermore, Benthey and colleagues employed a support vector machine-based model to analyze CT scans and predict the risk of hemorrhagic transformation following the administration of thrombolytics in ischemic stroke patients. The model accurately predicts the risk of hemorrhagic transformation, which can assist physicians in determining the optimal thrombolytic therapy for each patient (19).

In the realm of rehabilitation, the application of AI has been shown to benefit from the use of audiovisual means to increase patient motivation. Computer interface systems, such as Braine, ensure game adaptation on the basis of the patient's motor requirements and adjust difficulty levels as the disease progresses, facilitating gradual improvement in patient functionality. This process is achieved by translating brain activities into signals that control, for example, the mouse (20). Additionally, other areas of future exploration for AI include electronic health records (EHR), picture archiving and communication systems (PACSs), and radiology information systems (RIS) (13) (Table 2).

**Table 2:** The use of AI for diagnosis is based on the type of stroke and vascular involvement.

Type of stroke and vascular involvement	Use of AI observed
**Diagnosis of ischemic stroke due to occlusion of medium-sized vessels.**	An exhaustive analysis evaluated how AI algorithms perform in diagnosing obstructions in the middle cerebral artery in its M2 segment. It was found that, overall, these algorithms have a sensitivity of 64% and a specificity of 97%, with an area under the curve of 0.79. However, their current performance suggests they are more useful as additional confirmation tools than as independent tools for detecting these obstructions. The reported sensitivity might not be sufficient to reliably rule out the presence of an obstruction in the M2 segment. Additionally, all analyzed studies had a retrospective design, which could introduce biases. Furthermore, heterogeneity in sample sizes, inclusion criteria, algorithms, and imaging parameters also affected the results. There is a need for further innovations and the potential use of more advanced CT-Angiography techniques to improve AI performance in this area. Despite the potential of these algorithms, substantial improvement is required before considering their widespread implementation in clinical practice (21).
**Diagnosis of ischemic stroke due to occlusion of large vessels**	On the other hand, research found on cases of obstruction in major vessels shows promising results regarding the application of AI. The analyzed studies indicate that integrating AI into an early care system significantly reduces the intervals between diagnosis and thrombectomy (22–24). Furthermore, it has been demonstrated that the use of software designed to detect obstructions in large-caliber vessels improves both sensitivity and specificity in their identification (25), However, there is a study that concludes that the detection of occlusion of a large vessel may be limited depending on its location, which decreases sensitivity and specificity (26). Another highlighted benefit in the literature is the decrease in hospital stay time, effectively comparing the periods before and after the implementation of AI for detecting obstructions in large vessels through CT-Angiography (27). Finally, AI has demonstrated the ability to predict possible outcomes at thirty days, using the Modified Rankin Scale as a basis, like other studies employing the interpretation of CT-Angiographies (28).
**Diagnosis of hemorrhagic stroke.**	Convolutional Neural Networks, a branch of DL, are used to detect, segment, quantify, and analyze intracerebral hemorrhages (ICH), as well as to predict the risk of hematoma progression. Although the noncontrast CT scan is the primary neuroimaging tool for hemorrhagic stroke, the availability of its interpretation by radiologists or neuroradiologists is not always guaranteed. In such situations, Convolutional Neural Networks emerge as valuable tools. Some examples of software dedicated to lesion detection using noncontrast CT scan include Accipiolx, Aidoc Briefcase ICH, CuraRad-ICH, HealthICH, RAPID ICH, and Viz ICH (29–31).
	Likewise, it is recognized that the rupture of aneurysms, without proper monitoring, constitutes a significant cause of intracerebral hemorrhages. Evaluating the prognosis of rupture of these aneurysms is essential when treating patients diagnosed with or having a history of this malformation. Models such as XGBoost and Convolutional Neural Networks have demonstrated efficacy in prediction through the analysis of angiographic images (32, 33).
	To conclude, considering populations with limited resources, Salman, Saif, and collaborators developed the Hemorrhage Evaluation and Detector System for Underserved Populations (HEADS-UP), a high-precision (95.8%) and fast cloud-based model for the detection and evaluation of intracerebral hemorrhages. Using the Google Cloud VertexAI AutoML tool, this system proved to be accurate and efficient, emerging as a promising tool to provide early services in primary-level hospitals (34).

### 3.4 Use of AI to predict the risk of death following a stroke

The prognosis of individuals experiencing a stroke is a determinant of treatment and rehabilitation focus. However, algorithms studied thus far have not shown reliable or reproducible data both in research and in practice when used, primarily owing to limitations in sample size, software misclassification errors, and questionable clinical utility, among other variables. Therefore, Schwartz, Lihi, and colleagues conducted a systematic review on the performance of ML models created up to July 2022 for predicting mortality after a stroke. They aimed to identify risk factors related to a better or worse prognosis of the disease. The results revealed that the random forest (RF) model was the best predictor of one-month mortality, with an AUROC of 0.82. The logistic regression (LR) model was the best predictor of in-hospital death, with an AUROC of 0.90 and a positive predictive value of 0.97 at the expense of a relatively modest sensitivity of 81%. Overall, the ML algorithms demonstrated a favorable range of AUROCs for mortality prediction (0.67–0.98). On the other hand, among the 20 variables studied as risk factors for mortality after a stroke, age, elevated body mass index (BMI), and National Institutes of Health Stroke Scale (NIHSS) score at admission were identified, with the highest sensitivity (100%), specificity (90.9%), and AUROC (0.97%) among the variables (35).

Similarly, Lin CH and colleagues conducted a study of 21,463 patients to evaluate the performance of stroke prognosis models on the basis of statistics from ML and DL. They identified critical factors for predicting long-term mortality from routinely available intrahospital information. The final poststroke prognosis models (penalized Cox model based on statistics, random survival forest (RSF) model based on ML, and DeepSurv model based on DL) were developed via software such as “Scikit-Survival” (version 0.17.1), pycox (version 0.2.3), and R (version 4.0). According to statistical verification and other ML and DL algorithms, all models achieved high and consistent performance in predicting long-term mortality after a stroke, approaching a concordance index (C-index) of 0.8, with no significant differences between them (36).

Finally, not only has the scientific community focused on the diagnosis, treatment, and prognosis of pathology as pillars of mortality and poststroke quality of life, but rehabilitation is also a fundamental part of the outcome that can result from proper management of the three principles. This is why, as a branch of AI, robotics has also been involved in this evolution of new technologies. While it is believed that the first 6 months poststroke are the crucial period for functional capacity restoration, recent studies on chronic strokes have challenged this premise. Consequently, new intensive interventions aimed at neuromotor function have been developed, which can lead to significant improvements beyond the first year after experiencing a stroke. An example of an advance in this field is the randomized, controlled, multicenter clinical trial on robots in chronic stroke (VA ROBOTICS) conducted by the Veterans Health Administration and associated organizations, which uses the “MIT—manus” device, which is designed for upper limb rehabilitation (37). This, along with many other recently published studies, has demonstrated the capacity and vast potential for the exploration of AI.

### 3.5 Assistance from AI in certain specialties

AI is transforming medical diagnosis and decision-making. Its ability to identify patterns and relationships in large amounts of data allows physicians to better select patients, diagnose more accurately, choose the most appropriate treatment, and predict outcomes. Medical-surgical specialties such as neurosurgery, neuroradiology, and neurology are experiencing accelerated interest in the use of AI (Table 3).

**Table 3:** Application of AI in medical and surgical specialties.

Medical-Surgical specialty	Application of AI
**Neurosurgery.**	According to El-Hajj, Victor Gabriel, and colleagues, AI in surgery has led to a shift from the paradigm of “human vs. machine” to a “human and machine” approach. Among the approaches with the highest number of related articles were spine, endovascular, and neuro-oncology. Regarding the “Uses” of this tool, those that were particularly studied were: assistance in diagnosis and imaging acquisition. However, other uses included: assistance in the decision-making process, determination of surgery candidates, as well as those requiring urgent surgery or those with high surgical risk. On the other hand, this systematic review conducted from 1996 to July 2022 had a surprisingly predominant number of articles originating from the United States or Canada, while the contribution of developing countries was practically absent. This relationship can be attributed to the lack of resources in these parts of the world, as the lack of resources not only limits the importation of AI-driven technology but also the progress of research. Therefore, this raises significant concerns regarding resource distribution and hence global inequality (38).
**Neurology.**	According to Vinny, P.W. and colleagues, AI promises to revolutionize neurology in unimaginable ways. This is because over the last decade, ML and/or DL have been able to diagnose strokes from CT scan and Nuclear Magnetic-Resonances, detect papilledema and diabetic retinopathy from retinal evaluations, as well as interpret electroencephalograms (EEGs) for coma prognosis, detect seizures prior to stroke onset, predict the progression from mild to severe cognitive impairment, and classify neurodegenerative diseases. However, its validity must be determined through clinical trials conducted in humans; otherwise, it will become another innovative tool that quickly falls into obsolescence (39).
**Neuroradiology.**	According to Duong, Michael Tran, and colleagues, research on AI in image interpretation has grown exponentially in recent years. A recent study identified several clinical tasks that AI can perform, including workflow organization, lesion and anatomical segmentation, quality, safety, multimodal integration, and detection of various diseases such as epilepsy, multiple sclerosis, neurodegeneration, trauma-related injuries, among others. However, AI is still not widely used in clinical practice. This is because current AI systems are often considered “black boxes,” meaning it is difficult to understand how they arrive at their conclusions. For AI to be more accepted by medical professionals, it is necessary to improve explainability, which will allow medical professionals to assess its accuracy and reliability (40).

## 4 Discussion

This scoping review addresses how AI, a subdomain of computer science, has experienced exponential growth in various disciplines, highlighting its impact on the healthcare sector, with an emphasis on the clinical approach to stroke. Over the past decade, with the increasing burden of chronic diseases, AI has significantly influenced prediction, diagnosis, and medical treatment, improving the quality of care for both patients and healthcare professionals. In the pharmacological field, AI also promises to significantly reduce drug development times by increasing the understanding of drug bioavailability and establishing reliable biomarkers (9, 10).

In the healthcare field, AI has demonstrated substantial benefits in estimating treatment success probabilities, preventing complications, supporting diagnosis and treatment, and identifying pathologies. The use of mobile applications and technologies such as the internet of Medical Things supports patients' awareness of their illness, improving treatment adherence. Within the medical field, disciplines such as pathology and radiology have experienced notable advances through the development of AI systems. Although still in the research phase, examples such as the “Idx-DR system” and “Viz. AI” demonstrate the potential of AI in diagnosing diabetic retinopathy and stroke, respectively. Additionally, AI has contributed to the analysis of medical images, showing exceptional accuracy in anomaly detection (10, 11).

The use of AI for stroke prediction, the second leading cause of mortality worldwide, has been the subject of several studies. Methods such as extreme gradient boosting-based tree classifiers have proven effective in predicting ischemic stroke in hospitalized patients. Similarly, supervised and unsupervised learning models have been explored to improve prediction accuracy. AI has also revolutionized the diagnosis and treatment of stroke. From optimizing workflows through convolutional neural networks to identifying candidates for mechanical thrombectomy, AI has streamlined processes and improved diagnostic accuracy. Models such as “multimodal U-Net” and “ensemble echo state networks” have demonstrated high accuracy rates in stroke classification and treatment adherence improvement (11, 15, 17).

Thus, the application of AI extends to specific diagnoses, such as the detection of obstructions in medium and large cerebral arteries, as well as the identification of hemorrhagic strokes. Additionally, in the field of rehabilitation, AI has shown benefits in that it uses audiovisual media and computer interface systems to improve patient functionality. AI is clearly transforming medical-surgical specialties such as neurosurgery, neurology, and neuroradiology. Collaboration between humans and machines is highlighted in neurosurgery, whereas AI promises to revolutionize neurology by diagnosing various neurological conditions through advanced ML and DL techniques (38–40).

An inherent disadvantage of all DL methods lies in their lack of transparency and interpretability. For example, it is impossible to definitively define which specific image features are used by the model to determine the outcome. Another widespread drawback is that each existing DL system is currently being developed to address specific diagnostic issues. Although DL techniques can be used to assist specialists in decision-making, they cannot be employed to perform fully automated diagnoses and are thus incapable of completely replacing physicians. Despite the potential for automation in precision medicine, open research questions persist. Who bears responsibility for an incorrect decision or prediction made by an AI system? How are security elements incorporated into these systems? Additionally, the question arises as to how the economy will react to the decrease in jobs due to AI implementation (6).

The challenge of adopting AI algorithms in healthcare is multifaceted. First, physicians are skeptical of the use of AI, as it employs algorithms that are considered “black boxes” and requires thorough clinical validation to support its results. Second, healthcare providers must trust the effectiveness of the algorithms before their implementation, which involves solid clinical validation and justification. Moreover, patients' reluctance to use AI-based healthcare services without fully understanding their operation poses a significant challenge. The question of whether the healthcare sector is ready for full integration of AI remains a topic of debate (8).

Primary care serves as the first point of contact for many patients at risk of stroke. Integrating AI into this setting could enable earlier identification of high-risk individuals, personalized prevention strategies, and streamlined referrals to specialized care. Examples include using AI algorithms to analyze patient histories and biometric data to predict stroke risk or employing mobile applications to support long-term management. These applications remain underexplored and warrant further investigation.

## 5 Conclusion

This scoping review underscores the transformative potential of AI in stroke care, particularly in diagnosis and treatment. AI-driven tools have demonstrated high accuracy in identifying stroke types, predicting outcomes, and optimizing treatment workflows. However, significant gaps remain, especially in the integration of AI into primary care settings. The findings align with the study's objectives by mapping the current landscape of AI applications in stroke care and identifying research gaps. Specifically, the review highlights the predominance of AI use in acute care, with limited exploration of its applications in prevention and rehabilitation; the underrepresentation of primary care settings in AI research, despite their critical role in early detection and long-term management; and the need for further clinical trials to validate AI tools and promote their adoption in real-world healthcare settings. To maximize the benefits of AI in stroke care, future efforts should focus on expanding research on AI applications in primary care to enhance prevention and early diagnosis; conducting large-scale clinical trials to validate AI models and improve their transparency and explainability; and encouraging interdisciplinary collaboration between AI developers, healthcare providers, and policymakers to address barriers to adoption. AI represents a new horizon in stroke management, offering the potential to improve patient outcomes, reduce healthcare costs, and enhance the efficiency of care delivery. However, achieving this goal requires targeted efforts to address the identified gaps and limitations, ensuring equitable and effective integration of AI into healthcare systems.
